# The association between risk of bias and effect sizes of spinal manipulative therapy in low back pain trials: meta-epidemiological protocol

**DOI:** 10.1016/j.mex.2026.103895

**Published:** 2026-04-01

**Authors:** Justine Tilborghs, Annemarie de Zoete, Michiel de Boer, Tiziano Innocenti, Javier Muñoz Laguna, Bayden J McKenzie, Raymond Ostelo, Casper Nim, Jan Hartvigsen, Sidney M Rubinstein

**Affiliations:** a Department of Health Sciences, Faculty of Science and Amsterdam Movement Sciences Research Institute - Program Musculoskeletal Health, Vrije Universiteit Amsterdam, Amsterdam, Netherlands; b Institut Franco-Européen de Chiropraxie, Toulouse, France; c Department of General Practice, Erasmus MC University Medical Center, Rotterdam, Netherlands; d Department of Primary and Long-term Care, University Medical Center Groningen, Groningen, Netherlands; e GIMBE Foundation, Bologna, Italy; f Musculoskeletal Epidemiology Research Group, University of Zurich and Balgrist University Hospital, Zurich, Switzerland; g Epidemiology, Biostatistics and Prevention Institute (EBPI), University of Zurich, Zurich, Switzerland; h University Spine Centre Zurich (UWZH), Balgrist University Hospital, University of Zurich, Zurich, Switzerland; i Musculoskeletal Health and Wiser Health Care Units, School of Public Health and Preventive Medicine, Monash University, Melbourne, Australia; j Department of Epidemiology and Data Science, Amsterdam University Medical Centre, Vrije Universiteit, Amsterdam, Netherlands; k Medical Research Unit, Spine Centre of Southern Denmark, Lillebaelt Hospital, University Hospital of Southern Denmark, Middelfart, Denmark; l Department of Regional Health Research, University of Southern Denmark, Odense, Denmark; m Center for Muscle and Joint Health, Department of Sports Science and Clinical Biomechanics, University of Southern Denmark, Odense, Denmark; n Chiropractic Knowledge Hub, Odense, Denmark

**Keywords:** Risk of bias, Spinal manipulative therapy, Low back pain, Effect sizes, Randomized controlled trials, Meta-epidemiological study

## Abstract

Systematic error undermines the internal validity of randomized controlled trials (RCTs). Elucidating how risk of bias (RoB) domains distort effect estimates strengthens evidence trustworthiness. Low back pain (LBP) research provides a representative field for this investigation, with spinal manipulative therapy (SMT) serving as a widely utilized intervention.

**Objective:**

To explore the association between risk of bias domains and effect estimates of SMT treatment in LBP trials.

**Methods:**

RCTs from the Cochrane systematic reviews which have examined the effect of SMT for acute and chronic LBP will be included. The Cochrane RoB 2 tool will be used. The influence of RoB domains on effect estimates for pain intensity and physical functioning will be explored through univariable and multivariable meta-regression models. Models will be adjusted for confounders identified through a directed acyclic graph, including sample size, trial registration, country income level, and comparator type, while exploring interactions.

**Significance:**

This study builds upon research in exercise therapy, investigating whether the association between RoB and effect estimates is consistent across other non-pharmacological LBP interventions. Findings may help refine the application of GRADE on SMT, and improve evidence synthesis and decision-making for researchers and policymakers.

## Graphical abstract

**Figure:**
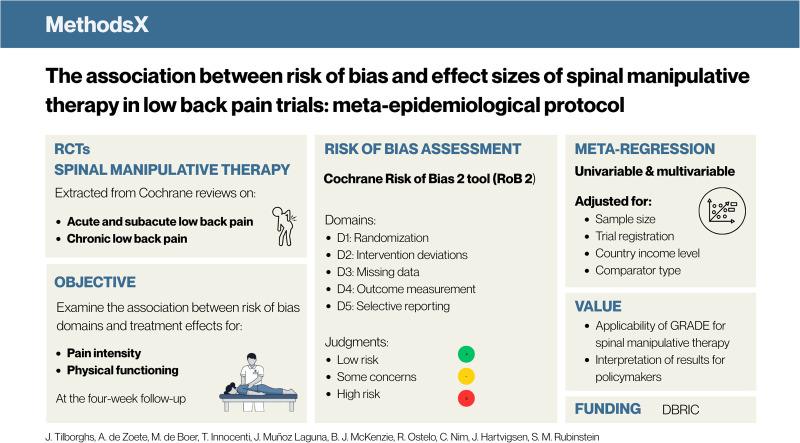

## Specifications table

**Table:** 

**Subject area**	Medicine and Dentistry
**More specific subject area**	Manipulation, Mobilization, Spinal Manipulative Therapy, Low back pain, Risk of bias
**Name of your protocol**	The association between risk of bias and effect sizes of spinal manipulative therapy in low back pain trials: meta-epidemiological protocol.
**Reagents/tools**	Not applicable.
**Experimental design**	Part 1: Risk of bias (RoB) assessment with the Cochrane RoB 2 tool. RCTs from the Cochrane systematic reviews which have examined the effect of SMT for acute (PMID: 22,972,127) and chronic (PMID: 21,328,304) LBP will be included. Part 2: The influence of RoB domains on effect sizes for pain intensity and physical functioning will be explored through univariable and multivariable meta-regression models. The multivariable models will be adjusted for a prespecified set of confounders (sample size, trial registration, country income level and intervention comparisons). To assess the robustness of the primary findings, sensitivity analyses will be conducted, and temporal trends for each RoB domain will be investigated.
**Trial registration**	Not applicable.
**Ethics**	This meta-epidemiological study does not require ethics approval as we will not be collecting personal data at the individual level. Instead, it will summarize information from publicly available RCT reports.
**Value of the Protocol**	• The primary objective is to assess whether RoB domains influence the outcomes ‘pain intensity’ and ‘physical functioning’ at the four-week follow-up. We will investigate how prespecified confounders account for RoB and the subsequent variation in methodological quality across spinal manipulative therapy (SMT) RCTs. • This study will determine if the impact of methodological bias on effect estimates is consistent across different non-pharmacological LBP interventions. • These findings may improve how the GRADE framework is applied to systematic reviews of SMT for low back pain. Furthermore, they may enhance the interpretation of these reviews by key stakeholders, such as researchers and policymakers.

## Background

Bias, defined as systematic error, directly undermines the internal validity and trustworthiness of randomized controlled trials (RCTs). Since bias cannot be measured directly, its potential must be rigorously assessed, referred to as risk of bias (RoB) [1,2].

Generally, as the RoB of an RCT increases, certainty in the results decreases. Previous meta-epidemiological research has demonstrated how specific methodological limitations in RCTs can lead to overestimated treatment effect estimates [1,3,4]. RoB assessment typically evaluates multiple domains, reflecting key aspects of study design and conduct. Fundamental shortcomings, such as inadequate allocation concealment and lack of blinding, are frequently associated with exaggerated treatment effects for medical interventions [2,[4], [5], [6]]. However, the association between specific RoB domains and effect sizes is not consistent [7,8].

Low back pain (LBP) warrants significant research attention given its substantial clinical and socioeconomic burden [[9], [10], [11]]. Among the various non-pharmacological interventions for LBP, there is still considerable uncertainty regarding how different RoB profiles influence reported treatment outcomes [12,13]. One such key intervention is spinal manipulative therapy (SMT), defined as a 'hands-on' treatment directed towards the spinal joints, including both spinal manipulation and mobilization techniques [14]. Since SMT is recommended as part of primary LBP management [14,15], some meta-epidemiological studies have investigated RoB in RCTs of SMT for LBP [[16], [17], [18]].

To assess the RoB, the Cochrane RoB 2 tool [4] may offer a more nuanced and robust evaluation compared to RoB 1. This study will extend previous research that examined the influence of methodological limitations on effect estimates for exercise therapy RCTs [19]. Given that SMT is fundamentally different from exercise therapy, it remains undetermined whether the previous results are replicable within SMT RCTs. It is particularly advantageous that RoB 2 offers a structured evaluation of blinding within domain 2 (deviations from intended interventions) and domain 4 (measurement of the outcome) for SMT RCTs, where blinding is rarely assessed and often difficult to achieve and maintain [20,21]. RoB 2′s signaling questions, algorithm and updated ratings (high/low/some concerns) may facilitate working through different challenges within the SMT context more effectively.

*Objective:* This study will explore the association between the RoB 2 ratings and treatment effect estimates from RCTs investigating SMT for acute, subacute and chronic LBP. We seek to determine whether the impact of methodological limitations on effect estimates is a general finding across non-pharmacological LBP interventions or is dependent on the intervention’s mechanism.

Findings may refine the application of the GRADE framework, specifically regarding RoB domains, in future systematic reviews on SMT interventions. This may enhance the reliability of evidence synthesis and facilitate better-informed decision-making for researchers and policymakers involved in the management of non-pharmacological LBP.

## Description of protocol

### Material and methods

**Study design.** This is a meta-epidemiological study that will explore the association between RoB domains and estimates of SMT treatment effects. The methodological approach has been adapted from a previous meta-epidemiological study for exercise therapy [22]. The study will be reported in adherence to the PRISMA-P (Preferred Reporting Items for Systematic Review and Meta-Analysis Protocols) statement [23]. We will also follow the adaptation of the PRISMA 2009 for meta-epidemiological studies proposed by Murad et al. [24].

## Data source

RCTs identified in the Cochrane review “Spinal manipulative therapy for acute and subacute low back pain” and “Spinal manipulative therapy for chronic low back pain” [25,26] will be included and the search will be updated.

## Data extraction

### Descriptive data

We will access descriptive study data previously extracted by two independent reviewers for RCTs from Cochrane reviews [25,26]. This includes study design characteristics, setting, year, country of conduct, population, interventions, comparisons, and outcome information. Complementary data, which are required for the application of the RoB 2 tool, will be extracted as needed.

### Assessment of risk of bias

We will use the revised Cochrane RoB tool for randomized trials, the RoB 2, to assess the risk of bias in included RCTs [27]. The evaluation will be facilitated by the dedicated Excel-based tool, available through the Cochrane RoB training materials on their website [28]. This Excel tool incorporates all required RoB 2 signaling questions, with the according answers (yes, probably yes, no information, probably no, no), and algorithms for systematic assessment.

To ensure high inter-rater reliability and consistency, the reviewer will complete a within-team training session based on the official Cochrane RoB 2 guidance and materials [27]. This training will include RoB assessments on a random selection of RCTs related to traction therapy for LBP. RoB assessments for SMT RCT for LBP will be performed by one reviewer (JT). These will be compared against RoB 2 assessments for the identical cohort of RCTs on High-Velocity Low-Amplitude (HVLA) spinal manipulation, previously conducted by a Danish research group for a separate publication [29]. The process was overseen by two co-authors of this protocol (CN, JH). Following the Cochrane Handbook [2], a study was at high RoB if any domain was scored as high risk or if 3 or more domains were having some concerns. A study was at low RoB if all domains were scored as low risk, and we will follow the same algorithm. Discrepancies identified between JT's assessments and the previous judgments will be resolved collaboratively by JT, CN and JH to achieve consensus. The RCTs on spinal mobilization not previously completed by the Danish team, but included in the Cochrane reviews of acute and chronic LBP, will be assessed independently by JML and BM, and a consensus will subsequently be sought with JT. A third reviewer (AdZ or SR) will be consulted for arbitration.

The RoB will be assessed for two outcomes separately (pain intensity and physical functioning), which are the most researched core domains in LBP [30]. The comprehensive dataset of detailed RoB judgments will be made available upon reasonable request to ensure transparency and reproducibility.

### Outcome variables

As this study utilizes descriptive data previously extracted for the Cochrane reviews on SMT for LBP, their outcome extraction hierarchies are respected. This is consistent with the Cochrane Handbook (section 18.4.1), supporting prioritization processes [6].

When an outcome is evaluated in a RCT using more than one scale, we will prioritize scales and standardize measurements as follows [31,32]:

• **Pain:** all pain intensity scores, regardless of the original scale used (e.g., Numerical Rating Scale [NRS], Visual Analogue Scale [VAS], or Pain severity subscale of the Brief Pain Inventory [BPI]), will be converted to a common 0 to 100-point scale. Linearly rescaling pain scores has shown to preserve relative differences comparative to those obtained from the original scales [33].
• **Physical functioning:** we will prioritize scales according to the following order: 1) Roland-Morris Disability Questionnaire (RMDQ); 2) Oswestry Disability Index (ODI); 3) Quebec Back Pain Disability Scale (QBPDS); 4) Any other back pain-specific scale.

**Effect sizes.** Pain will be examined as a mean difference (MD). To ensure comparability across different physical functioning scales, we will apply standardization based on an internal reference standard rather than study-specific standard deviations. In the absence of an external reference, this internal standard will be constructed using the average of the pooled standard deviations at baseline, following the worked example (Table 6.2) described by the NICE Guidelines Technical Support Unit [34]

**Time points.** Outcome data closest to the four-week follow-up will be used to maximize power, as one month is the most often reported time point in the included trials. Data collection at four weeks captures short-term effects while minimizing the attrition bias. If no data are available for this time point, we will use data from the three months follow-up time point that does not exceed 12 weeks. When an outcome measure is recorded in multiple ways during a relevant follow-up period (e.g., NRS during last week, NRS last 24 hours, NRS at rest, etc.), we will select one measure based on the following preference order and recall periods: 1) during last week; 2) during the last 24 hours; 3) at the moment (1. at rest, 2. at night, 3. anything else); and 4) any other time frame. This preference order will be established to ensure a consistent selection of the most clinically representative pain score, favouring measurements with an acceptable recall period that better captures the overall patient experience. Additionally, priority will be given to average pain, as it, theoretically, provides a more representative and stable measure of the overall treatment effect compared to extreme values like most severe or least pain.

### Risk of bias domains (Central determinant)

For each outcome, our central determinant of interest is the RoB rating, which will be calculated for each of the five domains using the algorithm set out by the RoB 2 developers [4]:

Domain 1: bias arising from the randomization process

Domain 2: bias due to deviations from intended interventions

Domain 3: bias due to missing outcome data

Domain 4: bias in the measurement of the outcome

Domain 5: bias in the selection of the reported result

The ratings for each domain within the RoB 2 tool are ‘high’, ‘low’, or ‘some concerns’, which will be categorized as a dichotomous variable ‘Low’ versus ‘High/Some concerns’ in order to maximize statistical power. Subsequently, an overall RoB judgment will be determined for each outcome, based on the integrated algorithms of the RoB 2 tool.

### Confounders

The following variables will be used as covariates:

• **Comparator type (categorical data):** 1) SMT versus sham SMT / placebo control (RCTs where a sham or placebo procedure was used as the control, regardless of the reported success of participant blinding); 2) SMT versus no treatment (participants received no treatment, a pamphlet only, or were on a waitlist); 3) SMT versus other conservative interventions (e.g. advice or education, usual care, exercise therapy, electrotherapy, psychological therapy, non-exercise multi-modal physical therapy, back school, relaxation, anti-inflammatories).

Following the Cochrane reviews [25,26], our analysis adopts their comparison types and classifies each included RCT accordingly, with a comprehensive overview of these classifications to be found in the original reviews.

When multiple contrasts from the same RCT will be examined in the same analysis (e.g. SMT versus sham and SMT versus no treatment), the number of participants in the SMT group will be divided by the number of comparators. This step corrects for errors introduced by 'double-counting' of participants in the same meta-analysis.

• **Sample size (discrete data):** Sample size plays a critical role in the size and precision of observed effects on pain or physical function. Smaller sample sizes lead to wider confidence intervals and greater uncertainty in effect estimates, making it harder to draw informative conclusions about treatment efficacy [26,35,36].
• **Prospective trial registration (dichotomous – yes/no):** ‘Yes’ indicates the study was prospectively registered and/or a complete study protocol is available. ‘No’ indicates the study was retrospectively registered or there was no registration or published protocol [22]. Trial registration will be included as in the multivariable meta-regression models to address potential residual confounding not fully captured by RoB domain 5. While domain 5 assesses selective reporting within a published article, it does not fully account for external biases related to the decision to publish the study, which prospective registration mitigates by fostering trialist accountability.
• **Country income level (categorical data):** The RCT location may be directly related to the level of funding, knowledge base and international collaboration, and can therefore have a significant impact on the methodological quality of the study. For this purpose, country income classifications will be determined using the most recent World Bank's official country income classifications available at the time of data analysis [37]. Classification using the income level at the time the trial was conducted seemed not feasible as the recruitment start year was inconsistently reported. The World Bank's 'low-income’, ‘lower-middle-income' and 'upper-middle-income' categories will be combined into a single 'low- and middle-income' group, while the 'high-income' category will remain distinct. This categorization aims to ensure sufficient sample sizes for robust analysis. The World Bank categories change over time but the only significant transition that could affect our dataset concerns countries moving between upper-middle and high-income status. We anticipate that this will likely have a negligible effect on the results.

### Rationale for confounder selection

The selection of these confounders is informed by existing meta-epidemiological literature, particularly the work of Innocenti et al. [19] on exercise RCTs, the CONSORT statement [38], and previous meta-epidemiological research within manual therapy [9,10,12,13,[16], [17], [18],[39], [40], [41]]. We developed a directed acyclic graph (DAG) using DAGitty software [42] to make explicit and prespecify our hypotheses about the network of important influences [43,44]. The complete DAG detailing all presumed causal pathways for all RoB 2 domains in the context of SMT RCTs is provided in Appendix A.1. A detailed rationale for the broader causal model is provided in Appendix A.2. The corresponding DAGitty model code is provided in Appendix A.3.

### Causal structure assumptions and elaboration

**Fig. 1:**
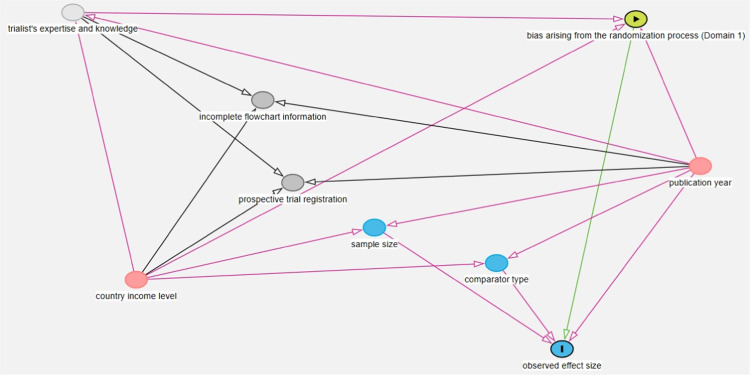
An illustrative diagram. Formalizing the authors assumptions about the causal structure between RoB domain 1 (bias arising from the randomization process) and treatment effects (outcome of interest) in RCTs of SMT for LBP.a.

The DAG helps to identify a minimal sufficient adjustment set for estimating the total effect of bias: sample size, comparator type, country income level, and prospective trial registration. The inclusion of these specific confounders in the adjustment set will be tailored to each RoB domain, reflecting their varying influence on the outcome. To facilitate comprehension of their role, we utilize an illustrative diagram of domain 1 only (bias arising from the randomization process) of the RoB 2 tool (Fig. 1).

Only one confounder (country income level) is assumed to directly influence domain 1. It is the single confounder to influence all five RoB domains, particularly as trialists in lower-income regions may be less equipped and subsequently less able to implement stringent reporting standards like CONSORT [45].

The other three confounders have no direct causal relationship with domain 1:

• Comparator type potentially influences the ‘observed effect size’. This risk primarily arises from performance (domain 2) and response bias (domain 4) dictating expectations regarding treatment effectiveness.
• Sample size is considered causally independent of domain 1; we assume the quality of the randomization process is determined by methodological rigor, which itself is reflective of the trialists' expertise, knowledge and commitment to robust study design, rather than the participant volume.
• Prospective trial registration documents researchers' intentions but does not directly influence the conduct of the randomization and allocation procedures evaluated in Domain 1.

The distinct pathways between the other RoB domains and the outcome measures differ from domain 1. The visual representation can be found in Appendix A.1, with the corresponding clarifications detailed in Appendix A.2.

## Data analysis

### Descriptive analysis

We will describe study design characteristics, setting, year, population, interventions, comparisons, and outcome information. We will descriptively report the results using numbers and percentages. We will visualize the RoB ratings using traffic light plots.

### Statistical analysis

All analyses will be performed using R software (R Foundation for Statistical Computing, Vienna, Austria, version 4.5.1).

For each meta-regression model performed, the underlying assumptions of linearity, homoscedasticity, independence of residuals, and normality of residuals will be checked.

Furthermore, the analyses will be conducted using the broad SMT category, as separate analyses for manipulation and mobilization are not feasible; many RCTs report these techniques inconsistently or inadequately, and the Cochrane review found no meaningful differences between SMT techniques [26].

*Assumption testing for sample size*: Prior to the primary univariate and multivariable analyses, we will evaluate whether sample size relates to the magnitude of treatment effect estimates in a linear or non-linear fashion (e.g., as a proxy for dissemination bias). If the best model fit is found to be non-linear, sample size will be included as a log-transformed covariate in all subsequent analytical steps to ensure a more accurate adjustment for this confounder.

*Step 3: Exploratory analyses*

*3.1. Investigation of temporal trends by rob domain*

We will examine the association between publication year (treated as raw continuous data) and the proportion of studies categorized as 'low risk of bias' for each RoB domain and overall RoB, and its potential influence on the pooled effect sizes for the primary outcome. This analysis will be performed using the:

• Initial RoB judgments
• Modified RoB judgments (as derived from step 1.3)

Given the potential influence of resource availability on trial conduct, we will examine the analyses separately for two distinct country income categories:

• Merged 'low- and middle-income' countries
• 'High-income' countries

*3.2. MetaForest analysis*

To further explore potential residual confounding, non-linear relationships and interactions among covariates, an exploratory MetaForest analysis will be conducted. This will help identify complex patterns that may not be captured by the previous analyses. The results of this MetaForest analysis will be treated as hypothesis-generating and will be used to complement the findings of the prespecified models in step 1 and step 2.

*Step 1: Primary meta-regression models:*

*1.1. Primary univariate models (Unadjusted rob effects)*

We will conduct separate meta-regression analyses for each outcome (pain intensity and physical functioning), with the treatment effect (MD or SMD between groups) as the dependent variable, and RoB as the central determinant. This risk will be dichotomously categorized as ‘Low’ versus ‘High/Some concerns’ for each of the five RoB 2 domains and for the overall RoB judgment. The associations derived from these models will represent unadjusted effect estimates, illustrating the isolated relationship between each RoB domain and the treatment effect per outcome without accounting for other variables.

*1.2. Primary multivariable models (Adjusted rob effects)*

Following the univariable analysis, the prespecified covariates visualized in the DAG will be included in multivariable meta-regression models. For the analyses of treatment comparison groups, dummy variables will be created with 'SMT versus no intervention' as the reference category. This will allow for the analysis of comparisons such as SMT vs. placebo and SMT vs. another intervention relative to this baseline comparison type. Separate models will be constructed for each of the five RoB 2 domains and for the overall RoB judgment. For each model, the RoB 2 domain (categorized as 'Low’ versus 'High/Some concerns') will serve as the primary central determinant.

Outcome (Difference in pain/physical functioning at 1 month) = *β*_0_ + *β*_1_ X_(RoB 2 domain,k)_ + *β*_2_ X_(treatment comparator,k)_+ *β*_3_ X_(sample size,k)_ + *β*_4_ X_(prospective trial registration,k)_ + *β*_5_ X_(country income level,k) +_ *μ*_k_ + ϵ_k_

The equation above will be adjusted by including only the confounders pre-specified in our DAG for each RoB domain.

*1.3. Modified rob judgment*

We acknowledge the inherent methodological challenge in blinding patients and caregivers in SMT RCTs [20,21]. This holds critical implications for our RoB assessment, as many RCTs are expected to be automatically classified as high RoB under domain 2 (bias due to deviations from the intended interventions) and domain 4 (bias in measurement of the outcome). Due to the nature of the group interventions, participants can often correctly identify their treatment assignment. This may influence the reported outcomes, as pain and physical functioning are patient-reported measures.

As this classification risks to overshadow the other signaling questions of the domains, we will override the domain judgment to omit the patient and caregiver blinding bias as follows:

• The final judgment for domain 2 will be based exclusively on the answers to signaling questions 2.6 (‘Was an appropriate analysis used to estimate the effect of assignment to intervention?’) and 2.7 (‘Was there potential for a substantial impact of the failure to analyze participants in the group to which they were randomized?’).
• The final judgment for domain 4 will be based exclusively on the answers to signaling questions 4.1 ((Was the method of measuring the outcome inappropriate?’) and 4.2 (‘Could measurement or ascertainment of the outcome have differed between intervention groups?’).
• The overall RoB judgment is overruled using the modified domain 2 and 4 assessments.

All meta-analyses mentioned above in 1.1 and 1.2 will be re-run using these new, modified, RoB judgments.

*Step 2: Sensitivity analyses*

To assess the robustness of our primary findings and explore their stability, we will conduct a replication of analyses with:

1. Alternative RoB categorization:

The studies rating ‘Some concerns’ will be regrouped with the ‘Low risk of bias’ instead of the ‘High risk of bias’ studies. We will re-run the analyses by using the RoB categorization as dummy variables with 'Low risk of bias' as the reference category (‘Low/Some concerns versus High’).

2. Dataset with outcome measurement time point at 4 weeks only:

Outcomes for the primary models are extracted at the time point closest to 4 weeks; time points up to 12 weeks are used as a backup where the 4 week measurement is unavailable. To investigate whether the findings are consistent, we will re-run the key models (from step 1.1, 1.2 and 1.3) on a distinct subgroup of articles for which data were exclusively extracted at a time point at 4 weeks only.

3. Alternative adjustment set including publication year:

For the applicable RoB domains, a secondary adjustment set from an alternative biasing path within the DAG will be employed. This needs the inclusion of publication year as a covariate to adjust for the time-dependent evolution of RCTs. Later publication years may reflect a period of more mature methodology in high-income countries, including improved study design and more resources to increase transparency in trial conduct. We will introduce a binary categorization of ‘publication year’ for studies published before or after 2010. This specific time point accounts for the broader dissemination of reporting guidelines and standards (i.e. publication data of the CONSORT checklist [38]).

## Protocol validation

Not applicable.

## Limitations

**1. Reliance on reporting quality for risk of bias assessment introduces a two-fold challenge:**

• Deficiencies in reporting, such as absence of crucial information or missing explanatory detail, necessitates a rating of ‘No information’ in the RoB 2 assessment. This may lead the RoB 2 algorithm to a domain judgment which may not accurately reflect the trial’s actual conduct. This can be resolved by seeking contact with authors when the methods are unclear, although this is dependent on availability and response of the authors.
• Discordance between publication and trial conduct: a trial may have been reported meticulously, fully adhering to standard reporting guidelines. However, the actual conduct of the trial in practice might have been flawed. This results in an unjustified positive domain judgment, as the RoB 2 tool can only reflect what is explicitly documented.

**2. Lack of discriminative ability**

If the overall reporting quality is highly uniform, the number of RCTs classified into different bias levels might be unbalanced. This limits the discriminative ability of the subsequent analyses to robustly explore whether different levels of bias are associated with variation in treatment effect sizes.

**3. Analytical choice regarding adjustment for double counting**

To adjust for double counting in studies with multiple contrasts, we will divide the SMT group by the number of comparators. We acknowledge that, in the random effects models we use, this may influence the relative weight of all studies and that it will increase the absolute weight of the study with multiple contrasts, as tau enters more than one line for the same study in the dataset [46]. However, only fourteen studies (16 %) in our dataset have multiple contrasts, so this will likely have a minimal effect on our results.

## Related research article

None.

## For a published article

None.

## Declaration of AI-assisted technologies in the writing process

Microsoft Copilot (2026) was used to assist in refining the wording of certain sections of this manuscript, including improving clarity, grammar, and phrasing.

## Funding

This study is supported by the Dutch-Belgian Research Institute of Chiropractic (DBRIC), located in Emmeloord, Netherlands (project number A.24-01). The funder had no role in the study design, data collection, data analysis, interpretation of results, writing of the manuscript, or the decision to submit this manuscript for publication.

## Supplementary material and/or additional information

Appendix A.1 - A directed acyclic graph (DAG)

Appendix A.2 - Causal model development and rationale

Appendix A.3 - DAG model code

## CRediT authorship contribution statement

**Justine Tilborghs:** Conceptualization, Methodology, Formal analysis, Writing – original draft, Writing – review & editing. **Annemarie de Zoete:** Conceptualization, Methodology, Formal analysis, Supervision, Writing – review & editing, Funding acquisition. **Michiel de Boer:** Methodology, Formal analysis, Writing – review & editing. **Tiziano Innocenti:** Conceptualization, Methodology, Formal analysis, Writing – review & editing. **Javier Muñoz Laguna:** Methodology, Formal analysis, Writing – review & editing. **Bayden J McKenzie:** Methodology, Formal analysis, Writing – review & editing. **Raymond Ostelo:** Conceptualization, Methodology, Formal analysis, Supervision, Writing – review & editing, Funding acquisition, Project administration. **Casper Nim:** Conceptualization, Methodology, Formal analysis, Supervision, Writing – review & editing, Funding acquisition. **Jan Hartvigsen:** Conceptualization, Methodology, Formal analysis, Supervision, Writing – review & editing, Funding acquisition. **Sidney M Rubinstein:** Conceptualization, Methodology, Formal analysis, Supervision, Writing – review & editing, Funding acquisition, Project administration.

## Declaration of competing interests

The authors declare that they have no known competing financial interests or personal relationships that could have appeared to influence the work reported in this paper.

## Data Availability

No data was used for the research described in the article.

## Appendix Supplementary materials

Image, application 1
