# Supplementary material for: The association between risk of bias and effect sizes of spinal manipulative therapy in low back pain trials: meta-epidemiological protocol
Source: MethodsX. 2026 Apr 1;16:103895. doi: 10.1016/j.mex.2026.103895 (PMC13091211; doi:10.1016/j.mex.2026.103895)
Supplement: Supplementary file 1 [file mmc1.docx]

**Appendix A.1.**

**A directed acyclic graph (DAG)**

This DAG visually represents the authors assumptions on the causal relationships between the Cochrane risk of bias 2 domains, potential confounders, and the observed effect sizes (for pain intensity and physical functioning) within spinal manipulative therapy trials.

For interpretation of the references to color in this figure legend, see DAGitty [[1]](https://www.zotero.org/google-docs/?gjEyNg)


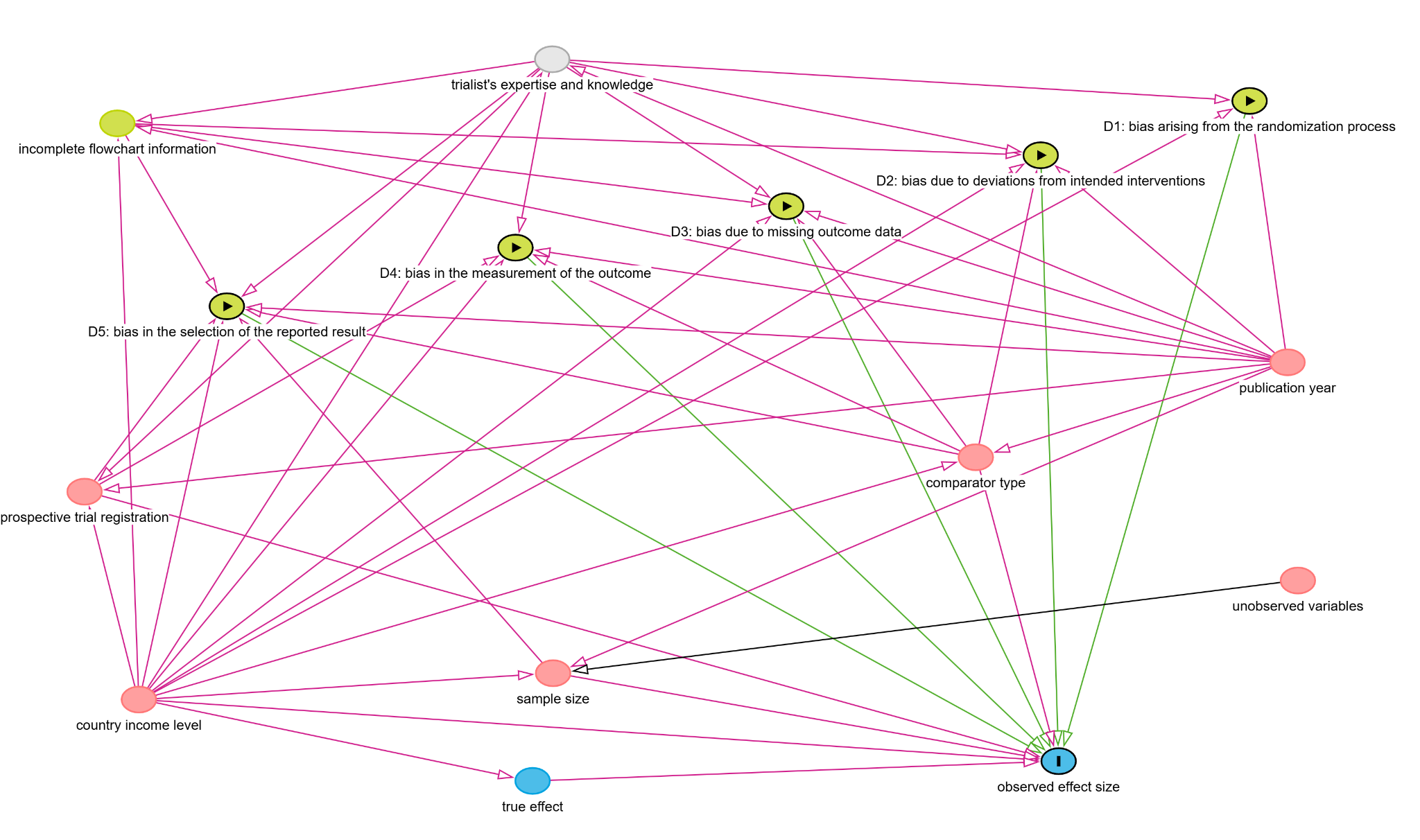


**Appendix A.2**. **Causal model development and rationale**

To formalize our causal hypotheses for spinal manipulative therapy RCTs, we developed a Directed Acyclic Graph (DAG) (Appendix A.1) following the ESC-DAG protocol [[1,2,3]](https://www.zotero.org/google-docs/?wyzB3i). The model integrates Innocenti's foundational DAG [[4]](https://www.zotero.org/google-docs/?qCAuJP) with meta-epidemiological findings in manual therapy [[5–14]](https://www.zotero.org/google-docs/?ahUZ1e), cross-referenced with established causal principles [[15,16]](https://www.zotero.org/google-docs/?lEixpF).

Model architecture:

- *Causal pathways:* Directed edges signify hypothesized causal effects. The 'observed effect size' reported in each RCT is driven by the 'true effect' (a latent variable) and influenced by all risk of bias (RoB) domains.
- *Trialist’s expertise and knowledge:* This unquantifiable cause influences multiple RoB domains [17]. To avoid multicollinearity, it is adjusted for through country income level and publication year.
- *Confounding:* Factors including sample size [8,18–20], comparator type [15], prospective trial registration [14,19,20], and country income level [15,21] are modeled as key confounders (see Fig. 1).
- *Reporting dynamics:* 'Incomplete flowchart information' impacts RoB domains 3, 4, and 5 [4,5]. Both participant flow reporting and prospective registration are correlated with publication year due to evolving reporting standards.
- *Variables omitted by design:* Other potential confounders, such as outcome type [15], were addressed through selection criteria and not depicted in the DAG. The influence of outcome measurement time points will be addressed via sensitivity analysis.

**REFERENCES**

[[1]](https://www.zotero.org/google-docs/?DSrxbU) [Textor J, Van der Zander B, Gilthorpe MS, Liśkiewicz M, Ellison GT. Robust causal inference using directed acyclic graphs: the R package ‘dagitty.’ Int J Epidemiol 2016;45:1887–94.](https://www.zotero.org/google-docs/?DSrxbU) https://doi.org/10.1093/ije/dyw341

[[2]](https://www.zotero.org/google-docs/?DSrxbU) [Ferguson KD, McCann M, Katikireddi SV, Thomson H, Green MJ, Smith DJ, et al. Evidence synthesis for constructing directed acyclic graphs (ESC-DAGs): a novel and systematic method for building directed acyclic graphs. Int J Epidemiol 2020;49:322–9. https://doi.org/10.1093/ije/dyz150.](https://www.zotero.org/google-docs/?DSrxbU)

[[3]](https://www.zotero.org/google-docs/?DSrxbU) [Watson ML, Hickman SH, Dreesbeimdiek KM, Kohler K, Stubbs DJ. Directed acyclic graphs in perioperative observational research–A systematic review and critique against best practice recommendations. Plos One 2023;18:e0281259.](https://www.zotero.org/google-docs/?DSrxbU) https://doi.org/10.1371/journal.pone.0281259

[[4]](https://www.zotero.org/google-docs/?DSrxbU) [Innocenti T, Hayden JA, Salvioli S, Giagio S, Piano L, Cosentino C, et al. Bias in the measurement of the outcome is associated with effect sizes in randomized clinical trials on exercise therapy for chronic low back pain: a meta-epidemiological study. J Clin Epidemiol 2023;162:145–55. https://doi.org/10.1016/j.jclinepi.2023.09.001.](https://www.zotero.org/google-docs/?DSrxbU)

[[5]](https://www.zotero.org/google-docs/?DSrxbU) [Hayden JA, Ellis J, Ogilvie R, Boulos L, Stanojevic S. Meta-epidemiological study of publication integrity, and quality of conduct and reporting of randomized trials included in a systematic review of low back pain. J Clin Epidemiol 2021;134:65–78. https://doi.org/10.1016/j.jclinepi.2021.01.020.](https://www.zotero.org/google-docs/?DSrxbU)

[[6]](https://www.zotero.org/google-docs/?DSrxbU) [Zambonin Mazzoleni G, Bergna A, Buffone F, Sacchi A, Misseroni S, Tramontano M, et al. A critical appraisal of reporting in randomized controlled trials investigating osteopathic manipulative treatment: a meta-research study. J Clin Med 2024;13:5181. https://doi.org/10.3390/jcm13175181.](https://www.zotero.org/google-docs/?DSrxbU)

[[7]](https://www.zotero.org/google-docs/?DSrxbU) [Niederer D, Weippert M, Behrens M. What modifies the effect of an exercise treatment for chronic low back pain? A meta-epidemiologic regression analysis of risk of bias and comparative effectiveness. J Orthop Sports Phys Ther 2022;52:792–802. https://doi.org/10.2519/jospt.2022.11149.](https://www.zotero.org/google-docs/?DSrxbU)

[[8]](https://www.zotero.org/google-docs/?DSrxbU) [Rubinstein SM, van Eekelen R, Oosterhuis T, de Boer MR, Ostelo RWJG, van Tulder MW. The risk of bias and sample size of trials of spinal manipulative therapy for low back and neck pain: analysis and recommendations. J Manipulative Physiol Ther 2014;37:523–41. https://doi.org/10.1016/j.jmpt.2014.07.007.](https://www.zotero.org/google-docs/?DSrxbU)

[[9]](https://www.zotero.org/google-docs/?DSrxbU) [Alvarez G, Solà I, Sitjà-Rabert M, Fort-Vanmeerhaeghe A, Gich I, Fernández C, et al. A methodological review revealed that reporting of trials in manual therapy has not improved over time. J Clin Epidemiol 2020;121:32–44. https://doi.org/10.1016/j.jclinepi.2020.01.006.](https://www.zotero.org/google-docs/?DSrxbU)

[[10]](https://www.zotero.org/google-docs/?DSrxbU) [Cashin AG, Lee H, Bagg MK, O’Hagan E, Traeger AC, Kamper SJ, et al. A systematic review highlights the need to improve the quality and applicability of trials of physical therapy interventions for low back pain. J Clin Epidemiol 2020;126:106–15. https://doi.org/10.1016/j.jclinepi.2020.06.025.](https://www.zotero.org/google-docs/?DSrxbU)

[[11]](https://www.zotero.org/google-docs/?DSrxbU) [de Almeida MO, Saragiotto BT, Maher C, Costa LOP. Allocation concealment and intention-to-treat analysis do not influence the treatment effects of physical therapy interventions in low back pain trials: a meta-epidemiologic study. Arch Phys Med Rehabil 2019;100:1359–66. https://doi.org/10.1016/j.apmr.2018.12.036.](https://www.zotero.org/google-docs/?DSrxbU)

[[12]](https://www.zotero.org/google-docs/?DSrxbU) [Savović J, Jones H, Altman D, Harris R, Jűni P, Pildal J, et al. Influence of reported study design characteristics on intervention effect estimates from randomised controlled trials: combined analysis of meta-epidemiological studies. Health Technol Assess 2012;16:1–82. https://doi.org/10.3310/hta16350.](https://www.zotero.org/google-docs/?DSrxbU)

[[13]](https://www.zotero.org/google-docs/?DSrxbU) [Arienti C, Armijo Olivo S, Ferriero G, Feys P, Hoogeboom T, Kiekens C, et al. The influence of bias in randomized controlled trials on rehabilitation intervention effect estimates: what we have learned from meta-epidemiological studies. Eur J Phys Rehabil Med 2023;60:135–44. https://doi.org/10.23736/S1973-9087.23.08310-7.](https://www.zotero.org/google-docs/?DSrxbU)

[[14]](https://www.zotero.org/google-docs/?DSrxbU) [Sénéquier A, Draper-Rodi J, Alvarez Bustins G, Braithwaite FA, Brown J, Corcoran D, et al. Investigating the trustworthiness of randomized controlled trials in osteopathic research: a systematic review with meta-analysis. J Clin Epidemiol 2025;183:111788. https://doi.org/10.1016/j.jclinepi.2025.111788.](https://www.zotero.org/google-docs/?DSrxbU)

[[15]](https://www.zotero.org/google-docs/?DSrxbU) Boutron I, Page MJ, Higgins JP, Altman DG, Lundh A, Hróbjartsson A. Chapter 7: Considering bias and conflicts of interest among the included studies [last updated August 2022]. In: Higgins JP, Thomas J, Chandler J, Cumpston M, Li T, Page MJ, et al, editor(s). Cochrane Handbook for Systematic Reviews of Interventions version 6.5. Cochrane, 2024. Available from: https://www.cochrane.org/authors/handbooks-and-manuals/handbook/current/chapter-07. Accessed 28 November 2025.

[[16]](https://www.zotero.org/google-docs/?DSrxbU) [Page MJ, Higgins JPT, Clayton G, Sterne JAC, Hróbjartsson A, Savović J. Empirical evidence of study design biases in randomized trials: systematic review of meta-epidemiological studies. PloS One 2016;11:e0159267. https://doi.org/10.1371/journal.pone.0159267.](https://www.zotero.org/google-docs/?DSrxbU)

[[17]](https://www.zotero.org/google-docs/?DSrxbU) Higgins JP, Savović J, Page MJ, Elbers RG, Sterne JAC. Chapter 8: Assessing risk of bias in a randomized trial [last updated October 2019]. In: Higgins JP, Thomas J, Chandler J, Cumpston M, Li T, Page MJ, et al, editor(s). Cochrane handbook for systematic reviews of interventions version 6.5. Cochrane, 2024. Available from: <https://training.cochrane.org/handbook/current/chapter-08>. Accessed 30 May 2025.

[[18]](https://www.zotero.org/google-docs/?DSrxbU) Higgins JP, Savović J, Page MJ, Elbers RG, Sterne JAC. [Chapter 13: Assessing risk of bias due to missing evidence in a meta-analysis](https://www.zotero.org/google-docs/?DSrxbU). Cochrane handbook for systematic reviews of interventions version 6.5. Cochrane, 2024. Available from: [https://www.cochrane.org/authors/handbooks-and-manuals/handbook/current/chapter-13 Accessed 29 July 2025.](https://www.zotero.org/google-docs/?DSrxbU)

[[19]](https://www.zotero.org/google-docs/?DSrxbU) Dechartres A, Trinquart L, Boutron I, Ravaud P. Influence of trial sample size on treatment effect estimates: meta-epidemiological study. BMJ. 2013 Apr 24;346:f2304. https://doi.org/10.1136/bmj.f2304.

[[20]](https://www.zotero.org/google-docs/?DSrxbU) [Dechartres A, Trinquart L, Faber T, Ravaud P. Empirical evaluation of which trial characteristics are associated with treatment effect estimates. J Clin Epidemiol 2016;77:24–37. https://doi.org/10.1016/j.jclinepi.2016.04.005.](https://www.zotero.org/google-docs/?DSrxbU)

[[21]](https://www.zotero.org/google-docs/?DSrxbU) [Medina CMS, Camacho CG, Moseley AM, Castellanos XT, Chen Q, Denova-Gutierrez E, et al. The methodological quality of clinical trials of physical therapy for low back pain varies between countries with different income levels. A meta-epidemiological study. Braz J Phys Ther 2024;28:101139.](https://www.zotero.org/google-docs/?DSrxbU) https://doi.org/10.1016/j.bjpt.2024.101139

**Appendix A.3. DAG model code**

| dag {  "D1: bias arising from the randomization process" [exposure,pos="0.302,-1.731"]  "D2: bias due to deviations from intended interventions" [exposure,pos="0.014,-1.439"]  "D3: bias due to missing outcome data" [exposure,pos="-0.284,-1.199"]  "D4: bias in the measurement of the outcome" [exposure,pos="-0.601,-1.004"]  "D5: bias in the selection of the reported result" [exposure,pos="-0.939,-0.728"]  "comparator type" [pos="-0.062,-0.017"]  "country income level" [pos="-1.042,1.124"]  "incomplete flowchart information" [pos="-1.129,-1.485"]  "observed effect size" [outcome,pos="0.035,1.414"]  "prospective trial registration" [pos="-1.157,-0.261"]  "publication year" [pos="0.411,-0.399"]  "sample size" [pos="-0.557,1.000"]  "trialist's expertise and knowledge" [latent,pos="-0.558,-1.891"]  "true effect" [pos="-0.581,1.507"]  "unobserved variables" [pos="0.370,0.576"]  "D1: bias arising from the randomization process" -> "observed effect size"  "D2: bias due to deviations from intended interventions" -> "observed effect size"  "D3: bias due to missing outcome data" -> "observed effect size"  "D4: bias in the measurement of the outcome" -> "observed effect size"  "D5: bias in the selection of the reported result" -> "observed effect size"  "comparator type" -> "D2: bias due to deviations from intended interventions"  "comparator type" -> "D3: bias due to missing outcome data"  "comparator type" -> "D4: bias in the measurement of the outcome"  "comparator type" -> "D5: bias in the selection of the reported result"  "comparator type" -> "observed effect size"  "country income level" -> "D1: bias arising from the randomization process"  "country income level" -> "D2: bias due to deviations from intended interventions"  "country income level" -> "D3: bias due to missing outcome data"  "country income level" -> "D4: bias in the measurement of the outcome"  "country income level" -> "D5: bias in the selection of the reported result"  "country income level" -> "comparator type"  "country income level" -> "incomplete flowchart information"  "country income level" -> "observed effect size"  "country income level" -> "prospective trial registration"  "country income level" -> "sample size"  "country income level" -> "trialist's expertise and knowledge"  "country income level" -> "true effect"  "incomplete flowchart information" -> "D2: bias due to deviations from intended interventions"  "incomplete flowchart information" -> "D3: bias due to missing outcome data"  "incomplete flowchart information" -> "D5: bias in the selection of the reported result"  "prospective trial registration" -> "D4: bias in the measurement of the outcome"  "prospective trial registration" -> "D5: bias in the selection of the reported result"  "prospective trial registration" -> "observed effect size"  "publication year" -> "D1: bias arising from the randomization process"  "publication year" -> "D2: bias due to deviations from intended interventions"  "publication year" -> "D3: bias due to missing outcome data"  "publication year" -> "D4: bias in the measurement of the outcome"  "publication year" -> "D5: bias in the selection of the reported result"  "publication year" -> "comparator type"  "publication year" -> "incomplete flowchart information"  "publication year" -> "prospective trial registration"  "publication year" -> "sample size"  "publication year" -> "trialist's expertise and knowledge"  "sample size" -> "D5: bias in the selection of the reported result"  "sample size" -> "observed effect size"  "trialist's expertise and knowledge" -> "D1: bias arising from the randomization process"  "trialist's expertise and knowledge" -> "D2: bias due to deviations from intended interventions"  "trialist's expertise and knowledge" -> "D3: bias due to missing outcome data"  "trialist's expertise and knowledge" -> "D4: bias in the measurement of the outcome"  "trialist's expertise and knowledge" -> "D5: bias in the selection of the reported result"  "trialist's expertise and knowledge" -> "incomplete flowchart information"  "trialist's expertise and knowledge" -> "prospective trial registration"  "true effect" -> "observed effect size"  "unobserved variables" -> "sample size"  } |
| --- |
